# Simplified Criteria to Assess Long-Term Antiviral Treatment Indication in Chronic HBV-Infected Pregnant Women in Cambodia

**DOI:** 10.3390/v16020194

**Published:** 2024-01-26

**Authors:** Jee-Seon Yang, Saren Sovann, Yusuke Shimakawa, Sovann Nhoueng, Bunnet Dim, Chanlina Vong, Channa Sann, Julia Guillebaud, Darapolin Vann, Bunrith Touch, Hyna Chea, Wathanak Pisey Choupoan Phirum, Eric Rosenthal, Christelle Paul, Leangchhun Khun, Chantana Yay, Denis Laurent, Samsorphea Chhun, Laurence Borand, Olivier Segeral

**Affiliations:** 1 Institut Pasteur du Cambodge, Phnom Penh 12201, Cambodia; jee.yang@aphp.fr (J.-S.Y.); jguillebaud@pasteur-kh.org (J.G.);; 2 Sorbonne Université, 75013 Paris, France; 3 Service de Pédiatrie Générale et Aval des Urgences, Hôpital Armand Trousseau, 75012 Paris, France; 4 Institut Pasteur, Unité d’Épidémiologie des Maladies Émergentes, Université Paris Cité, 75015 Paris, France; 5 Calmette Hospital, Phnom Penh 12201, Cambodia; 6 Jayavarman VII Hospital, Siem Reap 17259, Cambodiabunrithtouch@gmail.com (B.T.);; 7 ANRS|Maladies Infectieuses Emergentes, 75000 Paris, France; eric.rosenthal@inserm.fr (E.R.);; 8 Internal Medicine Department, Université Côte d’Azur, 06000 Nice, France; 9 Direction Department, Foundation Children’s Hospital Kantha Bopha, Phnom Penh 12000, Cambodia; 10 Center for Tuberculosis Research, Division of Infectious Diseases, Johns Hopkins University School of Medicine, Baltimore, MD 21205, USA; 11 HIV Unit, Infectious Diseases Department, Geneva University Hospital, 1205 Geneva, Switzerland

**Keywords:** hepatitis B, pregnancy, postpartum, long-term treatment, public health, international guidelines

## Abstract

Pregnant women identified to carry hepatitis B surface antigen (HBsAg) should be linked to care for the determination of the need for long-term antiviral therapy (LTT). We assessed the performance of simplified criteria, free from HBV DNA quantification, to select women eligible for LTT using different international guidelines as a reference. A retrospective analysis of HBV-infected pregnant women enrolled in the phase 4 ANRS TA-PROHM study was conducted in Cambodia. Sensitivity, specificity, and AUROC were computed to compare three simplified criteria (TREAT-B, HBcrAg/ALT, and TA-PROHM) with the American (AASLD) and European (EASL) guidelines as a reference. An additional assessment was performed at 6 months postpartum. Of 651 HBsAg-positive women, 209 (32%) received peripartum antiviral prophylaxis using tenofovir disoproxil fumarate (TDF). During pregnancy, 9% and 12% of women were eligible for LTT according to AASLD and EASL guidelines, respectively; 21% and 24% of women were eligible for prophylactic TDF and 2% and 5% in those ineligible (*p* < 0.001). Using the AASLD guidelines, the AUROC of TREAT-B, HBcrAg/ALT, and TA-PROHM scores were 0.88 (95%CI, 0.85–0.90), 0.90 (95%CI, 0.87–0.92), and 0.76 (95%CI, 0.73–0.80), respectively. Using the EASL guidelines, the AUROCs were lower: 0.73 (95%CI, 0.69–0.76), 0.76 (95%CI, 0.73–0.80), and 0.71 (95%CI, 0.67–0.74), respectively. Among those ineligible for prophylactic TDF, only 2% to 6% present an indication for LTT at 24 weeks postpartum. Few pregnant women are eligible for LTT, and the use of simplified criteria could represent an efficient triage option in decentralized areas to identify those negative for whom there is no urgent indication for LTT and focus on those positive for whom other exams must be conducted to confirm LTT indication.

## 1. Introduction

In 2019, chronic infection with hepatitis B virus (HBV) affected 296 million people and was responsible for 0.8 million deaths worldwide [1]. Approximately 70% of people living with HBV infection reside in areas with high endemicity, such as Southeast Asia, the Western Pacific, and Sub-Saharan Africa [1]. Mother-to-child transmission (MTCT) of HBV is the primary cause of chronic hepatitis B infection in countries with high endemicity, particularly in Southeast Asia, the Western Pacific, and Sub-Saharan Africa [2]. The World Health Organization (WHO) aims to achieve a 90% reduction in HBV incidence and a prevalence below 0.1% in children by 2030 [3]. All pregnant women are recommended to undergo screening for HBsAg, along with Human Immunodeficiency Virus (HIV) testing during pregnancy. If HBsAg is positive, HBV DNA levels are quantified. If the level is ≥5.3 log10 IU/mL, peripartum antiviral prophylaxis with tenofovir disoproxil fumarate (TDF) is recommended from the 28th week of pregnancy to delivery [4,5]. Peripartum antiviral prophylaxis can be discontinued either immediately after delivery or from 12 weeks postpartum, depending on the specific guidelines [4,5,6]. 

The assessment of liver diseases in HBsAg-positive women during pregnancy or the postpartum phase may present great opportunities to identify those who may benefit from long-term antiviral treatment (LTT) for their own health [5,7]. In all international guidelines, LTT is recommended in cases of ongoing viral replication associated with moderate or severe liver inflammation and/or fibrosis [5,6,7,8]. In the absence of HBV DNA quantification, liver biopsy, or liver stiffness measurement (LSM), implementing these recommendations is challenging in many low-income and middle-income countries (LMICs). In the 2015 guidelines, the WHO added a conditional recommendation to consider treatment based on persistently abnormal alanine aminotransferase (ALT) levels alone, defined by three elevated ALT measurements over a period of 6 to 12 months [9]. However, in LMICs, the majority of pregnant women are unaware of their HBV status, and therefore, no previous ALT measurements are available. In Gambia and Vietnam, the TREAT-B score, based on hepatitis B e antigen (HBeAg) and ALT levels, reported high accuracy in identifying HBV-infected individuals who require antiviral therapy compared to the simplified WHO treatment criteria and the Vietnamese guidelines [10,11]. Hepatitis B core-related antigen (HBcrAg) could also be a useful alternative to HBV DNA quantification: its strong correlation with serum HBV DNA levels was reported, irrespective of viral genotypes, in treatment-naïve patients with chronic HBV infection [12]. A rapid diagnostic test that detects HBcrAg (HBcrAg RDT) was recently developed. The sensitivity and specificity of HBcrAg RDT to diagnose HBV DNA levels were 72.7% and 91.7% for ≥2000 IU/mL, 86.7% and 88.7% for ≥20,000 IU/mL, and 91.4% and 86.3% for ≥200,000 IU/mL [13].

In Cambodia, the ANRS 12345 TA-PROHM study reported that an immunoglobulin-free strategy, using an HBeAg rapid diagnosis test (RDT) and ALT-based algorithm to assess eligibility for TDF prophylaxis, was effective in preventing MTCT when TDF was initiated at least four weeks before delivery [14]. The following algorithm was used: all HBV-infected pregnant women positive for HBeAg or with ALT ≥ 40 IU/L were eligible for TDF prophylaxis. This algorithm provided a sensitivity and specificity of 79.2% and 93.3%, respectively, to identify women with HBV DNA > 5.3 log10 IU/mL [15]. 

We aimed to assess, in this cohort of HBV-infected pregnant women, (1) the proportion of women in need of long-term antiviral treatment (LTT) for significant liver disease and (2) the accuracy of simplified criteria free from HBV DNA quantification and LSM to select women eligible for LTT, using the treatment criteria from different international guidelines as a reference.

## 2. Materials and Methods

### 2.1. Study Design

We conducted a retrospective analysis of all pregnant women enrolled in the ANRS 12345 TA-PROHM study (ClinicalTrials.gov, NCT02937779) after January 2019. This was a single-arm, multicenter, phase 4 trial conducted in five maternity units in Cambodia from 4 October 2017, to 27 November 2020. Until 31 December 2018, eligibility for prophylactic TDF was determined solely based on a positive HBeAg RDT during pregnancy. Starting 1 January 2019, the algorithm was expanded to include those who were HBeAg RDT-negative with ALT ≥ 40 IU/L [14]. The current analysis is restricted to women enrolled after the implementation of the latter algorithm.

### 2.2. Study Participants

Eligible participants were pregnant women aged 18 years or above with a positive HBsAg. Exclusion criteria included a positive serological test for HIV or hepatitis C virus (HCV), ongoing HBV treatment on the day of inclusion, creatinine clearance of less than 30 mL/min (according to the Cockcroft–Gault formula), severe gravid disease on the day of inclusion, evidence of a pre-existing fetal anomaly incompatible with life, or intention to deliver in a maternity outside of the study sites.

### 2.3. TA-PROHM Study Flow

First, pregnant women were screened with an SD BIOLINE HBsAg RDT (Standard Diagnostics [SD], INC., Suwon, Gyeonggi-do, Republic of Korea) during an antenatal care visit. Women positive for HBsAg RDT were further tested with SD BIOLINE HBeAg RDT (Standard Diagnostics [SD], INC., Suwon, Gyeonggi-do, Republic of Korea). HBV DNA quantification was performed using a quantitative Polymerase Chain Reaction (PCR) assay targeting the S gene of HBV (PUMA HBV kit, Omunis, Clapiers, France) according to the manufacturer’s instructions. AST/ALT levels were measured on ABX PENTRA C400 (Horiba, Kyoto, Japan) using the International Federation of Clinical Chemistry method (ultraviolet without pyridoxal phosphate). TDF prophylaxis was indicated for women positive for HBeAg RDT or with ALT ≥ 40 IU/L. Women negative for HBeAg RDT and with ALT < 40 IU/L were ineligible for prophylactic antivirals and did not receive TDF. HBV DNA quantification was retrospectively performed using sera obtained at inclusion but was not used to indicate eligibility for TDF prophylaxis. Women eligible for prophylactic therapy received 300 mg of TDF orally once a day from 24 weeks of gestation until 6 weeks postpartum. While women enrolled before 24 weeks of gestation were appointed for a week-24 consultation to start TDF, those enrolled at 24 weeks or later started TDF immediately. Participant characteristics (age, alcohol consumption, parity, term, known HBV status) were also retrieved at inclusion.

After delivery, maternal and infant visits were scheduled at 6 weeks and 6 months postpartum. At 6 weeks postpartum, all women, irrespective of their TDF prophylaxis eligibility during pregnancy, were invited to undergo liver disease examinations, including APRI score, LSM using transient elastography (FibroScan 402, Echosens, Paris, France), and liver ultrasound. For women receiving prophylactic TDF, a decision to continue or stop TDF was made by an external committee of clinicians within 2 weeks after liver disease assessment. The decision to continue TDF was based on the following criteria: suspected cirrhosis (according to the clinical, biological, ultrasound reports, and APRI score), HBV DNA > 4.3 log IU/mL & ALT > 2 ULN, HBV DNA > 3.3 log IU/mL & persistent ALT > 2 ULN (2 measures separated by at least one month) & LSM > 7 kPa, family history of liver cancer, or new pregnancy planned in the next year. The last study visit was planned at 6 months postpartum. Mothers gave a last blood sample at this study visit, which was used to detect HBeAg RDT and measure HBV DNA and ALT/AST concentration.

Retrospectively, women’s plasma stored at −80 °C were screened for HBcrAg using an RDT (Fujirebio Inc., Tokyo, Japan) [13].

### 2.4. International Guidelines

Guidelines from the American Association for the Study of Liver Disease (AASLD) and the European Association for the Study of Liver (EASL) were reviewed and summarized in Table S1. These guidelines largely depend on factors such as cirrhosis, HBV DNA levels, HBeAg status, ALT, and fibrosis staging using liver histopathology or LSM [4,5,7,8,16]. We applied the Upper Limit Normal (ULN) for ALT recommended by each guideline. For fibrosis staging, we defined significant fibrosis (≥F2) and cirrhosis (F4) with the LSM level recommended by the guidelines when addressed. AASLD did not recommend any LSM threshold, so we applied ≥7 kPa and ≥11 kPa for significant fibrosis (≥F2) and cirrhosis (F4), respectively, after a review of the literature [17]. For the purpose of this study, we decided not to take into account the family history of liver cancer to assess the performance of the criteria. This is because this criterion could be independent of biological parameters and fibrosis staging.

### 2.5. Simplified Criteria

We assessed the performance of four simplified criteria to identify women eligible for LTT according to AASLD and EASL guidelines [4,5,7,8]. The first simplified criterion was the TREAT-B score, which utilized HBeAg status and ALT levels. The score was obtained by adding HBeAg (1 point if positive) and ALT levels: <20 IU/L (0 points), 20–39 IU/L (1 point), 40–79 IU/L (2 points), ≥80 IU/L (3 points). The TREAT-B score ranged from 0 (HBeAg-negative and ALT < 20 IU/L) to 4 (HBeAg positive and ALT ≥ 80 IU/L) [10]. The second was the TA-PROHM algorithm, utilizing positive HBeAg RDT and/or ALT ≥ 40 IU/L [14,15]. The third was the positivity of HBcrAg RDT, and the fourth was a score obtained by adding HBcrAg RDT (1 point if positive or double positive), and ALT levels: <20 IU/L (0 point), 20–39 IU/L (1 point), 40–79 IU/L (2 points), ≥80 IU/L (3 points). HBcrAg-based scores were only available at inclusion as it was only tested on samples at that moment of the study [13].

Eligibility for LTT and performances of the simplified criteria to identify women in need of LTT were assessed:

1. At inclusion, according to AASLD and EASL guidelines.
2. At week 24 postpartum, among TDF-ineligible women, according to AASLD & EASL guidelines.

### 2.6. Statistical Analysis

Descriptive statistics were used to summarize baseline patient characteristics. *p*-values were obtained by using Mann–Whitney–Wilcoxon non-parametric tests for continuous variables in the absence of normal distribution (Shapiro–Wilks test). For categorical variables, fisher exact tests were used.

The area under the receiver operating characteristic (AUROC), the sensitivity, and the specificity were calculated for the different scores and with three different cutoff points for TREAT-B and HBcrAg/ALT scores.

Statistical analysis was performed using STATA, version 17 (Statacorp College Station, College Station, TX, USA).

## 3. Results

**Figure 1:**
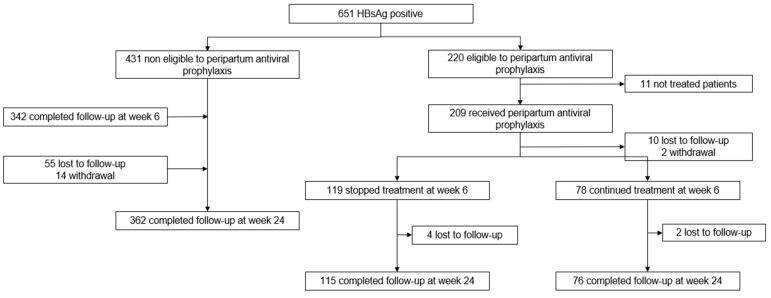
Flow-Chart of the Ta Prohm Study Population from 1 January 2019.

From 4 October 2017 to 17 December 2019, 21,251 pregnant women were screened for HBsAg, and 1194 patients were HBsAg positive. Of those, 651 were included from 1 January 2019 and enrolled in the study. Thirty-four percent (220/651) were eligible for TDF prophylaxis, and 95% (209/220) effectively received it (Figure 1).

### 3.1. Characteristics at Inclusion

Women’s characteristics at inclusion are reported in Table 1. Overall, the median age was 29 years old (IQR, 25–33), the median term of gestation at inclusion was 22 weeks (IQR, 20–27), and 31% (199/651) were aware of their HBV status. Women eligible for TDF prophylaxis were significantly younger (*p* < 0.001) and had higher HBV DNA levels (*p* < 0.001).

During pregnancy, according to AASLD, 9% (56/651) were eligible for LTT overall, 21% (46/220) among those eligible for TDF prophylaxis vs. 2% (10/431) for those ineligible for TDF prophylaxis (*p* < 0.001). According to EASL, 12% (75/651) were eligible for LTT overall, 24% (53/220) among those eligible for TDF prophylaxis vs. 5% (22/431) for those ineligible for TDF prophylaxis (*p* < 0.001).

### 3.2. Performance of Simplified Criteria at Inclusion

The performances of the simplified criteria at inclusion are detailed in Table 2.

Using AASLD as a reference, the performance of TREAT-B, HBcrAg/ALT and TA-PROHM scores to identify women for LTT indication were very good with an AUROC at 0.88 (95%CI, 0.85–0.90), 0.90 (95%CI, 0.87–0.92) and 0.76 (95%CI, 0.73–0.80), respectively. Using the TA-PROHM score, the sensitivity was 82.1%, and the specificity was 70.8%. For the two other scores, a cutoff of ≥2 had a higher sensitivity (80.7% for TREAT-B, 87.5% for HBcrAg/ALT) but a lower specificity (79.2% for TREAT-B and 75.3% for HBcrAg/ALT). A cutoff of ≥3 had a higher specificity (98.7% for TREAT-B and 97.0% for HBcrAg/ALT) but a lower sensitivity (51.8% for TREAT-B and 62.5% for HBcrAg/ALT). Using the EASL as a reference, the performances of TREAT-B, HBcrAg/ALT and TA-PROHM scores to identify women for LTT indication were lower with an AUROC at 0.72 (95%CI, 0.69–0.76), 0.76 (95%CI, 0.72–0.79) and 0.71 (95%CI, 0.67–0.74), respectively.

The characteristics of women with false negative results are described in Table 3. Overall, the majority of women ineligible according to the simplified criteria but eligible to AASLD or EASL guidelines have ALT < 40 UI/L but HBV DNA > 3.3log10/mL (median, 6.53 log10/mL (IQR 3.98–6.96)) and LSM > 7 kPa (median, 9.85 (IQR 7.5–12.1)).

### 3.3. Postpartum Evaluation

Among women eligible for prophylactic TDF (n = 220), 90% (197/220) were assessed at week 6, and 40% (78/197) effectively pursued TDF as a LTT (Figure 1). Among those ineligible for prophylactic TDF (n = 431), 84% (362/431) completed the evaluation at week 24 postpartum. Postpartum characteristics are described in Table 4.

Among them, 6% (22/362) and 6% (21/362) were eligible for LTT at week 24, according to AASLD and EASL, respectively. Ten and nine women had an LSM suggestive of late-stage severe fibrosis according to AASLD and EASL guidelines, respectively, but none of them had clinical or biological signs of cirrhosis. Among those eligible for LTT according to the AASLD criteria, 55% (12/22) were also eligible for EASL. LSM results were involved in the decision to pursue TDF in 41% (9/22) of women eligible for AASLD guidelines and 90% (19/21) with EASL guidelines (Table 5).

## 4. Discussion

Our study reports that 9% and 12% of HBV-infected pregnant women are eligible for LTT according to AASLD and ESAL guidelines, respectively: 21% and 24% in women eligible for TDF prophylaxis and 2% and 5% in those ineligible for TDF prophylaxis, respectively. Using the AASLD guidelines as a reference, the performances of TREAT-B, HBcrAg/ALT and TA-PROHM scores to identify women in need of LTT during pregnancy were good with an AUROC at 0.88 (95%CI, 0.85–0.90), 0.90 (95%CI, 0.87–0.92) and 0.76 (95%CI, 0.73–0.80), respectively. Using the EASL guidelines, the performances of these same scores were lower with an AUROC at 0.73 (95%CI, 0.69–0.76), 0.76 (95%CI, 0.73–0.80) and 0.71 (95%CI, 0.67–0.74), respectively. Among the women ineligible for TDF prophylaxis, very few (6%) presented indications for LTT at 24 weeks postpartum, according to AASLD & EASL guidelines.

To our knowledge, this is the first study analyzing the indications for LTT in a large cohort of HBV-infected pregnant women in Asia. In this population of young women (median age 29 years old), only 10% of them needed LTT. The ANRS 12345 TA-PROHM study was conducted in five hospitals in the country, of which three were located in the province. Of those, one hospital (Foundation of Children’s Hospitals Kantha Bopha) attracts women from urban and rural areas coming from all over Cambodia. Consequently, pregnant women enrolled in the ANRS 12345 TA-PROHM study could be considered representative of the Cambodian population. This data is reassuring regarding the severity of liver disease in this age group compared to data for older populations in neighboring countries [11]. We found that women eligible for TDF prophylaxis were more likely to be also eligible for LTT. The algorithm validated in the ANRS 12345 TA-PROHM study to determine eligibility for TDF prophylaxis has been developed to identify HBeAg-negative women with elevated HBV DNA levels at risk of MTCT; they are known for carrying pre-core mutations and thus at risk of cytolysis and significant fibrosis [18,19]. The use of such algorithms could, therefore, serve a dual purpose by enabling the identification of those eligible for LTT who would require a more sustained follow-up and those who would not require it and for whom the liver disease assessment could be delayed.

TA-PROHM algorithm’s sensitivity to detect women in need of LTT according to AASLD and EASL guidelines is 82% and 71%, respectively. The HBcrAg RDT/ALT score has a higher sensitivity, 87%, with AASLD guidelines but a lower, 61%, with EASL guidelines. For all these scores, the false negative women are mainly women with low ALT levels (<40 IU/L) but with an HBV DNA >3.3 logIU/mL and an elasticity ranging from 6 to 8 kPa. The LSM thresholds to define significant fibrosis differ from one guideline to another, ranging from 6 kPa for EASL to 7 kPa for AASLD and 8 kPa for APASL. The lower threshold of LSM in EASL guidelines partially explains the highest number of false negative women. Uncertainty about these intermediate thresholds makes decision-making complicated in the absence of consensus [20]. Another reason is related to the eligibility of HBeAg-positive women aged more than 30 years old with an HBV DNA >5.3 logIU/mL whatever the ALT level in EASL guidelines, which is not part of AASLD guidelines. For all these women, the LTT initiation is probably not an emergency, and subsequent liver disease assessment 6 to 12 months later seems a reasonable option. In populations where the eligibility for LLT is close to 10%, the simplified criteria have a 98% negative predictive value to exclude LTT indication. These simplified criteria could represent a triage option to identify negative women for whom the vast majority have no urgent indication for LTT and for whom no rapid, specific liver assessment seems to be necessary for the postpartum phase. This hypothesis seems to be confirmed in our study as only 6% of women not eligible for peripartum antiviral prophylaxis are eligible for LTT at 6 months postpartum. While new WHO guidelines are still pending and Chinese guidelines recommend treating all patients with detectable HBV DNA and persistently elevated ALT levels [21], the paradigm is to shift toward simplified criteria. Using rapid diagnosis tests (HBeAg RDT and HBcrAg RDT) and ALT level, these criteria free from HBV DNA quantification can be easily integrated into decentralized care in rural areas. By using capillary blood with no requirement for electricity or centrifugation with an operating temperature of up to 39 °C and a rapid turnaround time (45 min), HBcrAg RDT could be particularly of interest for use in LMICs.

However, when using these simplified criteria, a large proportion of HBV-infected pregnant women might be unnecessarily treated. HBV treatment is currently life-long and requires regular monitoring with potential toxicity, which is problematic for young women if the indication is unclear. For women positive with the simplified criteria, other exams are necessary to identify those really in need of LTT. While liver biopsy is out of context in the absence of accessibility to the pathology department, LSM could represent an interesting option. In this study, 41% to 88% of the eligible population for LTT are due to LSM results, and 1.5% of women had an LSM suggestive of cirrhosis, but none of them had clinical, biological, or ultrasound signs in favor of this diagnosis. The use of LSM to detect patients with severe fibrosis or cirrhosis seems consensual (>11–12 kPa) in AASLD and EASL guidelines. However, in a large population of patients with chronic liver disease of various etiologies, comparing liver stiffness measurement and fibrosis stage assessed on liver biopsies, a cutoff value of 17.6 kPa had negative and positive predictive values for the diagnosis of cirrhosis of 92% and 91%, respectively [22]. Similarly, in patients with chronic viral hepatitis B or C, patients with LSM values of ≥17 kPa had a clinically significant incidence of liver-related complications, while patients with LSM values <17 kPa were not associated with adverse outcomes [23]. A consensual decision regarding LSM values for the diagnosis of significant fibrosis and significant cirrhosis would be beneficial. Overall, consistency among international guidelines is confounded by their complexity and differences in set points [24]. The forthcoming WHO guidelines must simplify and standardize LTT indication and improve the expansion of programmatic access to testing and treatment.

Our study has some limitations. The use of an external committee to decide TDF continuation for TDF-eligible women instead of stopping for all after delivery, as currently recommended, prevented us from having a consistent overall assessment for all postpartum women. The ANRS 12345 TA-PROHM study was designed and started before these recommendations, and the risk of severe flare-ups in cases of significant fibrosis carried weight at that time. The second limitation is that LSM was only performed in postpartum and not at inclusion as it was not recommended during pregnancy at the time of the conduct of the study. Additionally, LSM was performed with no food restrictions, and women with LSM above normal were not offered a fasting LSM control.

## 5. Conclusions

In Cambodia, approximately 10% of pregnant women appear to be eligible for long-term antiviral treatment (LTT). Simplified criteria could serve as an efficient triage option in decentralized areas, helping identify those without an urgent indication for LTT and directing attention to those who test positive, warranting further examinations to confirm the need for LTT. Utilizing rapid diagnostic tests (HBeAg RDT and HBcrAg RDT) along with ALT levels, these criteria, free from HBV DNA quantification, can be seamlessly integrated into decentralized care in rural areas.

## Figures and Tables

**Table 1:** Characteristics of the patients at inclusion.

	Overall Population (n = 651)		Eligible for Prophylactic TDF (n = 220)		Ineligible for Prophylactic TDF (n = 431)		*p*-Value
Age, years	29	(25–33)	27	(24–32)	30	(26–34)	<0.001
Alcohol consump.	3	0%	1	0%	2	0%	0.74
Primiparas	194	30%	75	34.09%	119	27.61%	0.09
Gestational age (WA)	22	(20–27)	22	(19–25)	23	(20–28)	0.37
Known HBV status	199	31%	58	29%	141	32.71%	0.10
HBeAg positive	151	23.20%	151	69%	0	0.00%	<0.001
ALT (IU/L)							
≥25	233	36%	114	55%	119	26.86%	<0.001
≥40	94	14%	94	43%	0	0.00%	<0.001
HBV viral load							
Median, IQR	3.35	(1.95–6.89)	7.81	(5.47–8.44)	2.53	(1.95–3.76)	<0.001
<3.3	322	49	33	15%	289	67%	
[3.3; 4.3[	83	13	12	5%	71	16%	
[4.3; 5.3[	38	6	9	4%	29	7%	
≥5.3	208	32	166	75%	42	10%	<0.001
APRI score							
1.5	14	2%	10	2%	4	1%	0.004
2.0	9	1%	6	3%	3	1%	0.044
TREAT-B score							
Median, IQR	1	(0–2)	2	(2–2)	0	(0–1)	<0.001
0	247	38%	0	0%	247	57%	
1	235	36%	51	23%	184	43%	
2	132	20%	132	60%	0	0%	
3	34	5%	34	15%	0	0%	
4	3	0%	3	1%	0	0%	<0.001
Eligibility for LTT							
By AASLD	56	9%	46	21%	10	2%	<0.001
By EASL	75	12%	53	24%	22	5%	<0.001

Data are presented in n (%) or median (IQR); Abbreviations: AASLD: American Association for the Study of Liver Disease, ALT: Alanine Aminotransferase, APRI: AST to Platelet Ratio Index, BMI: Body Mass Index, Consump: consumption, EASL: European Association for the Study of the Liver, Fam. Hist of liver K: Familial history of liver cancer, HBV: Hepatitis B Virus, TDF: Tenofovir Disoproxil Fumarate; TREAT-B: Treatment Eligibility in Africa for the Hepatitis B Virus; WA: Weeks of Amenorrhea.

**Table 2:** Comparison of AUROC for the performance of simplified criteria to select women eligible for the long-term antiviral treatment at antenatal examination (n = 651).

	HBcrAg Only	HBcrAg + ALT			TREAT-B (HBeAg + ALT)			TA-PROHM
Cutoff	NA	≥2	≥3	4	≥2	≥3	4	NA
AASLD 2018								
AUROC	0.71 (0.68–0.75)	0.90 (0.87–0.92)			0.88 (0.85–0.90)			0.76 (0.73–0.80)
TP	40	49	35	10	46	29	3	46
FP	172	147	18	0	124	8	0	174
TN	423	448	577	595	471	587	595	421
FN	16	7	21	46	11	27	53	10
Sen	71.4	87.5	62.5	17.9	80.7	51.8	5.4	82.1
	(57.8–82.7)	(75.9–94.8)	(48.5–75.1)	(8.9–30.4)	(68.1–90.0)	(38.0–65.3)	(1.1–14.9)	(69.6–91.1)
Spe	71.1	75.3	97.0	100.0	79.2	98.7	100.0	70.8
	(67.3–74.7)	(71.6–78.7)	(95.3–98.2)	(99.4–100.0)	(75.7–82.4)	(97.4–99.4)	(99.4–100.0)	(66.9–74.4)
EASL 2017								
AUROC	0.76 (0.73–0.80)	0.76 (0.72–0.79)			0.72 (0.69–0.76)			0.71 (0.67–0.74)
TP	60	46	24	9	39	21	3	53
FP	152	150	29	1	130	16	0	167
TN	424	426	547	575	446	560	576	409
FN	15	29	51	66	36	54	72	22
Sen	80.0	61.3	32.0	12.0	52.0	28.0	4.0	70.7
	(69.2–88.4)	(49.4–72.4)	(21.7–43.8)	(5.6–21.6)	(40.2–63.4)	(18.2–39.6)	(0.8–11.2)	(59.0–80.6)
Spe	73.6	74.0	95.0	99.8	77.4	97.2	100.0	71.0
	(69.8–77.2)	(70.2–77.5)	(92.8–96.6)	(99.0–100.0)	(73.8–80.8)	(95.5–98.4)	(99.4–100.0)	(67.1–74.7)

Abbreviations: AASLD: American Association for the Study of Liver Disease, ALT: Alanine Aminotransferase, AUROC: Area Under the Receiver Operating Characteristic, EASL: European Association for the Study of the Liver, FN: False Negative, FP: False Positive, Sen: Sensitivity, Spe: Specificity, TDF: Tenofovir Disoproxil Fumarate, TN: True Negative, TP: True Positive, TREAT-B: Treatment Eligibility in Africa for the Hepatitis B Virus.

**Table 3:** Characteristics of false negative women with AASLD and EASL criteria at an antenatal examination.

Simplified Criteria	Characteristics	N	%
AASLD 2018			
**Ta—Prohm** HBeAg - & ALT < 40 IU/mL	LSM > 7 & PCR HBV > 3.3	10	100
**Treat-B** HBeAg - & ALT < 40 IU/ mL HBeAg + & ALT < 20 IU/mL	LSM > 7 & PCR HBV > 3.3 LSM > 11	9 2	82 18
**Score AgHBcr** HBcrAg - & ALT < 40 IU/mL HBcrAg + & ALT < 20 IU/mL	LSM > 7 & PCR HBV > 3.3 LSM > 11 & PCR HBV < 3.3	5 2	71 29
EASL 2017			
**TA—PROHM** HBeAg - et ALT < 40 IU/mL	6 < LSM < 7 & PCR HBV > 3.3 LSM > 7 & PCR HBV > 3.3 LSM > 11 & PCR HBV < 3.3	9 12 1	41 55 5
**Treat-B** HBeAg - & ALT < 40 IU/ mL HBeAg + & ALT < 20 IU/mL	LSM < 6 & PCR HBV > 5.3 & Age > 30 6 < LSM < 7 & PCR HBV > 3.3 LSM > 7 & PCR HBV > 3.3 LSM > 11 & PCR HBV < 3.3	8 13 14 1	22 36 39 3
**Score AgHBcr** HBcrAg - & ALT < 40 IU/mL HBcrAg + & ALT < 20 IU/mL	LSM < 6 & PCR HBV > 5.3 & Age > 30 6 < LSM < 7 & PCR HBV > 3.3 LSM > 7 & PCR HBV > 3.3 LSM > 11 & PCR HBV < 3.3	8 10 10 1	28 34 34 3

Abbreviation: Unit: LSM (kPa), age (years) & PCR HBV (log10/mL).

**Table 4:** Characteristics of women at week 24 (n = 362).

	Ineligible for Prophylactic TDF (n = 342 for Fibroscan, n = 362 *)		Eligible for Prophylactic TDF (n = 202 **)		Overall Population (n = 564 ***)	
Characteristic	N or Median	IQR or%	N or Median	IQR or%	N or Median	IQR or%
Fibroscan (kPa) 1	4.1	3.4–5.0	4.4	3.4–5.0	4.2	3.5–5.1
Success Rate	100	92–100%				
>6 kPa	36	11%	30	15%	66	12%
>7 kPa	18	5%	13	6%	31	6%
>8 kPa	11	3%	7	3%	18	3%
Platelet ^1^ (G/L)	269	231–314	274	228–331	272	231–322
HBV DNA viral load (log IU/mL) ^2^						
Median, IQR	1.95	1.95–3.57	4.57	2.01–7.62	2.99	1.95–4.59
<3.3	253	70%	65	32%	85	15%
[3.3; 4.3[	56	16%	29	14%	38	7%
[4.3; 5.3[	23	6%	15	7%	118	21%
≥5.3	27	8%	91	45%	5	1%
ALT (IU/L)						
≥25, n (%)	207	57%	152	75%	359	64%
≥40, n (%)	62	17%	82	41%	144	26%
TREAT-B score						
Median, IQR	1	1–1	2	1–3	1	1–2
0	88	24%	5	2%	93	17%
1	212	59%	51	25%	263	47%
2	51	14%	81	40%	132	23%
3	11	3%	49	24%	60	11%
4	0	0%	15	7%	15	3%
Eligibility to LTT	22	6%	44	22%	66	12%
HBeAg Seroconversion	0	0%	7 ****	3%	7	1%

Data are presented in n (%) or median (IQR); ALT: Alanine Aminotransferase, HBV: Hepatitis B Virus, TDF: Tenofovir Disoproxil Fumarate, TREAT-B: Treatment Eligibility in Africa for the Hepatitis B Virus. * 55 lost to follow-up & 14 withdrawal, ** 17 lost to follow-up & 1 withdrawal, *** 72 lost to follow-up & 15 withdrawal **** 7 among 151 HBeAg-positive patients, thus 5%, ^1^ data retrieved at week 6 (n = 539), ^2^ 3 missing data.

**Table 5:** Eligibility for long-term treatment at week 24 postpartum (n = 568).

	Characteristics	Ineligible for Prophylactic TDF (n = 362 *)		Eligible for Prophylactic TDF (n = 202 **)	
AASLD	LSM = F4	5	23%	5	11%
	HBeAg-pos & DNA ≥ 4.3	0	0%	31	70%
	HBeAg-pos & DNA ≥ 4.3 & N < ALT ≤ 2N & LSM = F2	0	0%	1	2%
	HBeAg-neg & DNA ≥ 3.3 & ALT > 2N	13	59%	7	16%
	HBeAg-neg & DNA ≥ 3.3 & N < ALT ≤ 2N & LSM = F2	4	18%	0	0%
	**TOTAL**	**22**	**6%**	**44**	**22%**
EASL	LSM = F4	5	24%	4	9%
	HBV DNA > 4.3 & ALAT > 2N	2	10%	13	30%
	HBV DNA > 3.3 & LSM = F2	14	67%	12	28%
	HBeAg-pos & HBV DNA > 4.3 & age > 30 years old	0	0%	14	33%
	**TOTAL**	**21**	**6%**	**43**	**21%**

* AASLD: ALT ULN = 25 IU/L, F2 = 7 kPa, F4 = 11 kPa; ** ALT ULN = 40 IU/L, F2 = 6 kPa, F4 = 12 kPa if ALT = ] ULN − 5 × ULN [ or 9 kPa if ALT ≤ ULN.

## Data Availability

The study data will not be publicly available because they are sensitive health data that could compromise the confidentiality of the patients included. This is in accordance with French and European legislation on clinical research and personal health data. Nevertheless, the research dataset that underlies the results reported in this Article, including deidentified participant data and data dictionary, can be made available through a request to the Scientific Advisory Board and to the French National Agency for Research on AIDS and Viral Hepatitis. The access request, if validated, will be framed by an agreement between the sponsor and the applicant. Additional documents, including a French translation of the protocol, statistical analysis plan, and informed consent forms, can also be made available. The dataset and the additional documents will be available immediately after publication and could be shared with researchers who provide a methodologically sound proposal after agreement from the Scientific Advisory Board and the French National Agency for Research on AIDS and Viral Hepatitis. Proposals should be submitted to the corresponding author.

## Supplementary Materials

The following supporting information can be downloaded at https://www.mdpi.com/article/10.3390/v16020194/s1, Table S1: Summary of adapted international guidelines; Table S2: Performance of simplified algorithms to select women eligible for long-term antiviral treatment 6 months after delivery among women ineligible for TDF prophylaxis (n = 362).
