# Supplementary material for: Simplified Criteria to Assess Long-Term Antiviral Treatment Indication in Chronic HBV-Infected Pregnant Women in Cambodia
Source: Viruses. 2024 Jan 26;16(2):194. doi: 10.3390/v16020194 (PMC10891875; doi:10.3390/v16020194)
Supplement: Supplementary file 1 [file viruses-16-00194-s001.zip › viruses-2794399-supplementary.pdf]

Supplementary Table S1: Summary of adapted international guidelines

<table>
<tr><td colspan="3">AASLD 2018</td></tr>
<tr><td>ALT ULN: 25 IU/mL</td><td>F2: NSP
F2 used: 7kPa</td><td>F4: NSP
F4 used: 11kPa</td></tr>
<tr><td colspan="3">● If Ag HBe positive:
○ ALT > 2 x ULN & HBV DNA ≥ 4·3 log 10/mL
○ ALT = 1-2 ULN & HBV DNA ≥ 4·3 log 10/mL & fibrosis ≥ F2
● If AgHBe negative:
○ ALT > 2 x ULN & HBV DNA ≥ 3·3 log 10/mL
○ ALT = 1-2 ULN & HBV DNA ≥ 3·3 log 10/mL & fibrosis ≥ F2
● Compensated F4 & HBV DNA ≤ 3·3 log 10/mL
● Decompensated F4</td></tr>
<tr><td colspan="3">APASL 2015</td></tr>
<tr><td>ALT ULN : 40 IU/mL</td><td>F2: 8kPa</td><td>F4: 11kPa</td></tr>
<tr><td colspan="3">● Compensated cirrhosis & HBV DNA > 3·3 log 10/mL
● Decompensated cirrhosis & detectable HBV DNA
● If AgHBe positive :
○ & HBV DNA > 4·3 log 10/mL & ALT > 2 x ULN (1 month)
○ & HBV DNA > 4·3 log 10/mL & LSM ≥ F2
● ALT > ULN & LSM ≥ F2
● If AgHBe negative
○ & HBV DNA > 3·3 log 10/mL & ALT > 2 x ULN (1 month)
○ & HBV DNA > 3·3 log 10/mL & LSM ≥ F2
● Age > 35 years & LSM ≥ F2</td></tr>
<tr><td colspan="3">EASL 2017</td></tr>
<tr><td>ALT ULN : 40 IU/mL</td><td>F2: 6 kPA</td><td>F4: 12 kPa if ALT =]ULN-5xULN[
or 9kPa if ALT ≤ ULN</td></tr>
<tr><td colspan="3">● Compensated or decompensated cirrhosis & detectable HBV DNA
● HBV DNA > 4·3 log 10/mL & ALT > 2 x ULN
● HBV DNA > 3·3 log 10/mL & moderate liver necro-inflammation or fibrosis
● AgHBe positive & high level of HBV DNA*& age > 30 years</td></tr>
</table>

ALT: Alanine Aminotransferase; AASLD: American Association for the Study of Liver Disease; APASL: Asian Pacific Association for the Study of the Liver; ASL: European Association for the Study of the Liver; F2 : significant liver fibrosis, F4 : cirrhosis, NSP : Non Specified in the guidelines ; ULN : Upper Limit of Normal, * HBV DNA non specified in the guidelines, decision to use 20,000 as the threshold

Supplementary Table S2. Performance of simplified algorithms to select women eligible for long-term antiviral treatment 6 months after delivery among women ineligible for TDF prophylaxis (n = 362)

	TREAT-B			Ta - Prohm	WHO
Cut-off	≥ 2	≥ 3	4	NA	NA
AASLD 2018					
AUROC	0·79 (0·75-0·83)			0·75 (0·70-0·79)	0·68 (0·63-0·73)
TP	14	2	/	14	14
FP	48	9	/	48	94
TN	292	331	/	292	246
FN	8	20	/	8	8
Sen	63·6	9·1	NA	63·6	63·6
Spe	85·9	97·4	NA	85·9	72·4
EASL 2015					
AUROC	0·54 (0·48-0·59)			0·51 (0·46-0·56)	0·62 (0·57-0·67)
TP	4	2	/	4	11
FP	58	9	/	58	97
TN	283	332	/	283	244
FN	17	19	/	17	10
Sen	19·1	9·5	NA	19·1	52·4
Spe	83.0	97·4	NA	83·0	71·6

Abbreviations: AASLD : American Association for the Study of Liver Disease; AUROC : Area Under the Receiver Operating Characteristic; EASL : European Association for the Study of the Liver; FN : False Negative; FP : False Positive; Sen : Sensitivity; Spe : Specificity; TN : True Negative, TP : True Positive
